# Trends and Seasonality of Emergency Department Visits and Hospitalizations for Suicidality Among Children and Adolescents in the US from 2016 to 2021

**DOI:** 10.1001/jamanetworkopen.2023.24183

**Published:** 2023-07-19

**Authors:** Youngran Kim, Trudy Millard Krause, Scott D. Lane

**Affiliations:** 1 Department of Management, Policy & Community Health, School of Public Health, The University of Texas Health Science Center at Houston; 2 Department of Psychiatry and Behavioral Sciences, McGovern Medical School, The University of Texas Health Science Center at Houston

## Abstract

**Importance:**

The detection of seasonal patterns in suicidality should be of interest to clinicians and US public health officials, as intervention efforts can benefit by targeting periods of heightened risk.

**Objectives:**

To examine recent trends in suicidality rates, quantify the seasonality in suicidality, and demonstrate the disrupted seasonality patterns during the spring 2020 COVID-19–related school closures among US children and adolescents.

**Design, Setting, and Participants:**

This population-based, descriptive cross-sectional study used administrative claims data from Optum’s deidentifed Clinformatics Data Mart Database. Participants included children aged 10 to 12 years and adolescents aged 13 to 18 years who were commercially insured from January 1, 2016, to December 31, 2021. Statistical analysis was conducted between April and November 2022.

**Exposures:**

Month of the year and COVID-19 pandemic.

**Main Outcomes and Measures:**

Rates and seasonal patterns of emergency department (ED) visits and hospitalizations for suicidality.

**Results:**

The analysis included 73 123 ED visits and hospitalizations for suicidality reported between 2016 and 2021. Among these events, 66.1% were reported for females, and the mean (SD) age at the time of the event was 15.4 (2.0) years. The mean annual incidence of ED visits and hospitalizations for suicidality was 964 per 100 000 children and adolescents (95% CI, 956-972 per 100 000), which increased from 760 per 100 000 (95% CI, 745-775 per 100 000) in 2016 to 1006 per 100 000 (95% CI, 988-10 024 per 100 000) in 2019, with a temporary decrease to 942 per 100 000 (95% CI, 924-960 per 100 000) in 2020 and a subsequent increase to 1160 per 100 000 (95% CI, 1140-1181 per 100 000) in 2021. Compared with January, seasonal patterns showed peaks in April (incidence rate ratio [IRR], 1.15 [95% CI, 1.11-1.19]) and October (IRR, 1.24 [95% CI, 1.19-1.29]) and a nadir in July (IRR, 0.63 [95% CI, 0.61-0.66]) during pre–COVID-19 years and 2021. However, during the spring of 2020, which coincided with school closures, seasonal patterns were disrupted and April and May exhibited the lowest rates.

**Conclusions and Relevance:**

The findings of this study indicated the presence of seasonal patterns and an observed unexpected decrease in suicidality among children and adolescents after COVID-19–related school closures in March 2020, which suggest a potential association between suicidality and the school calendar.

## Key Points

**Question:**

Did trends and seasonal patterns of suicidality among children and adolescents change after the onset of the COVID-19 pandemic in March 2020?

**Findings:**

This cross-sectional study of 73 123 emergency department (ED) visits and hospitalizations for suicidality found that the incidence of ED visits and hospitalizations increased from 2016 to 2021, with a temporary decline in 2020. Prior to the pandemic, monthly incidences were typically higher during the school year, but during the spring of 2020, coinciding with school closures, they were substantially lower.

**Meaning:**

This study’s findings suggest that the unexpected decrease in suicidality among children and adolescents after school closures supports hypotheses that suicidality is associated with the US school calendar.

## Teaser

This cross-sectional study uses administrative health claims data for emergency department (ED) visits and hospitalizations to examine recent trends in suicidality rates, quantify the seasonality in suicidality, and demonstrate the disrupted seasonality patterns during the spring 2020 COVID-19–related school closures among US children and adolescents.

## Introduction

Suicidal ideation refers to thoughts about or a preoccupation with killing oneself, while suicide attempts involve actual acts of self-harm with the intent to die.^1^ Suicidality encompasses both suicidal ideation and suicide attempts and is strongly predictive of youth suicide deaths and warrants attention. It is more prevalent among adolescents than adults, and several studies have revealed increasing trends in adolescent suicidality over the past decades.^2,3,4,5^ In the US National Comorbidity Survey, 12.1% of adolescents reported serious thoughts of suicide, and approximately one-third of those with suicidal ideation attempted suicide before reaching adulthood.^6^ Rates of emergency department (ED) visits for intentional self-harm in 2020 were highest among adolescents aged 15 to 19 years compared with older age groups (472 vs 133 per 100 000),^2^ and youth suicidal ideation or suicide attempt rates in the US nearly doubled from 2008 to 2015.^5^

Seasonal patterns in suicidality should interest clinicians and US public health officials, as intervention efforts can benefit by targeting periods of heightened risk.^7^ There are several hypotheses focused on exogenous variables associated with seasonal suicidality patterns, including temperature changes,^8,9^ circadian rhythms, sunlight exposure,^8,9,10^ geographic latitude, and interactions with gender, substance use, and mental health status.^8,9,10,11,12^ However, these hypotheses have not focused on unique differences across age groups despite evidence that adolescence represents a developmental period of increased risk for suicidality.^13,14,15,16,17^ Some literature suggests that peaks and nadirs in adolescent suicidality may be associated with the school calendar near the start and end of the school year, with corresponding decreases during the summer months when school is out of session.^5,14,17,18,19,20,21,22^ These studies suggest that children and adolescents can face increased stress and decreased mental health when school is in session. Some known risk factors, such as school bullying and peer pressure about alcohol and drug abuse,^23,24,25,26,27,28^ may be associated with the observed seasonal patterns in suicidality during the school year. The in-school experience brings social, academic, and extracurricular stressors as well as poorer sleep habits, each of which may have detrimental outcomes for child and adolescent mental health and well-being.^29,30^

However, some uncertainty exists regarding the association of the in-school experience with suicidality patterns because similar peaks, typically in the spring, are also well documented across the adult life span.^13,18,31,32,33,34,35,36^ In this study, we hypothesized that the spring 2020 COVID-19–related school closures, which occurred uniformly across the US, would disrupt the seasonality of suicidality, deviating from the typical seasonal patterns. Specifically, we expected that the usual increase in suicidality during the spring months would not be observed during the school closures. Given the inherent challenge of disambiguating the association of in-school variables from other exogenous factors associated with suicidality patterns, leveraging disruption in school attendance can serve as a valuable “natural experiment.”^37^

Recent studies indicated that changes in suicidality were associated with the COVID-19 lockdown and school closures both in Europe^38,39,40^ and the US.^41,42^ However, the available data were often limited to specific hospital-based information, focused on the early months of 2020, or restricted to suicide attempts or deaths. Also, epidemiologic data on the rates of suicidal ideation and suspected suicide attempts are limited. Many surveillance data or studies did not include cases of suicidal ideation or direct hospitalization and reported suicidality as the percentage of total ED visits for suspected cases or observed number of ED visits from selected hospitals instead of incidence rates per population at risk.^5,17,43^

This study provides rates of child and adolescent suicidality as ED visits and hospitalizations for suicidal ideation and suicide attempts per population at risk focusing on 3 aims: (1) to examine temporal trends in suicidality rates over the most recent time frame for which nationally representative data were available, (2) to quantify the seasonality in suicidality by monthly variations, and (3) to demonstrate the disrupted seasonality patterns in suicidality during the spring 2020 COVID-19–related school closures as evidence for the potential association between seasonality patterns in suicidality and the academic calendar.

## Methods

### Study Design and Study Population

This study was a population-based cross-sectional analysis using Optum’s Clinformatics Data Mart derived from a database of administrative health claims for members of large commercial and Medicare Advantage health plans. The database includes approximately 17 million to 19 million annual covered lives for a total of more than 47 million unique lives during the study period from January 1, 2016, to December 31, 2021, representing geographically diverse populations spanning all 50 states. We included children aged 10 to 12 years and adolescents aged 13 to 18 years who had enrollment records during the measurement month and year, serving as the denominator population for calculating incidence rates. On average, there were approximately 1.2 million children and adolescents enrolled each year during the study period. To ensure ethical considerations, the study protocol was reviewed by The University of Texas Health Science Center at Houston institutional review board, which granted a waiver of informed consent due to the use of deidentified data. This study followed the Strengthening the Reporting of Observational Studies in Epidemiology (STROBE) reporting guideline.

### Outcome Measures

Study outcomes included temporal trends in monthly and annual rates of ED visits and hospitalization for suicidality among children and adolescents from 2016 to 2021. The unit of analysis was the ED visit or hospitalization; if an ED visit resulted in consecutive hospitalization, it was counted as a single event. We identified suicidality cases from medical claims files using the *International Statistical Classification of Diseases, Tenth Revision, Clinical Modification* (*ICD-10-CM*) codes for suicidal ideation and intentional self-harm or suicide attempts requiring ED visits or hospitalizations (eTable 1 in Supplement 1).^41,44,45^ We reported intentional self-harm and suicide attempts collectively (referred to as “suicide attempts”) following many of the national systems for suicide prevention surveillance that combine reports of suicide attempts and intentional self-harm.^17,41,46,47,48^ Although attempted suicide and deliberate self-harm that is not suicidal in nature are very different behaviors from a psychiatric perspective, the two are often blurred together from an epidemiologic injury surveillance perspective, and self-injurious behavior, particularly behavior requiring acute medical attention, is often reported as a suicide attempt.^5,48^ Also, the current *ICD-10-CM* codes related to intentional self-harm do not distinguish between self-harm with intent to die and self-harm with no intent to die.^46^ Although there is a code labeled “suicide attempt” (T14.91) in the *ICD-10-CM*, coding guidelines restrict the assignment of this code to cases in which the mechanism of the suicide attempt is unknown, and the National Center for Health Statistics categorizes this code as unspecified self-harm in the *ICD-10-CM* External Cause-of-Injury Framework.^45,46,49^ If there were *ICD-10-CM* codes indicating more than 1 condition, we determined the category exclusively based on a hierarchal classification, with suicide attempt being the highest priority to avoid double counting.

### Statistical Analysis

Statistical analysis was conducted between April and November 2022. Temporal trends were reported as rates per 100 000 enrollees by month and year estimated from Poisson regressions adjusting for age, sex, and region to account for differences in denominator populations unless they were stratified. For annual rates, denominator populations were weighted by the number of enrolled months during the measurement year. Estimated rates were obtained using the StataMP, version 17 (StataCorp LLC) *margins* command after the Poisson regression. For seasonal variations, we excluded 2020 data as we observed the disruption in seasonality patterns, particularly in the early COVID-19 pandemic when social distancing and school closures were implemented. To visualize seasonality, we plotted percentage deviations of estimated monthly rates from the mean expected monthly rates for overall incidence, subcategories of suicidal ideation and suicide attempts, and subgroups of males, females, children, and adolescents. To quantify seasonality, we reported the incidence rate ratio (IRR) and 95% CI in reference to January using Poisson regressions adjusting for age, sex, census region, and yearly trends. To illustrate the disruptions in seasonality patterns during the school closure period, we overlaid the monthly rates for the years 2019, 2020, and 2021. We also conducted a sensitivity analysis to assess whether similar disruptions were observed among older age groups. Significance levels were set at *P* < .05 for 2-tailed tests, and all analyses were performed using StataMP, version 17.

## Results

### Characteristics of ED Visits and Hospitalizations for Suicidality

There were 73 123 ED visits and hospitalizations for suicidality from 2016 to 2021 among children and adolescents. Two-thirds of these events (66.1%) were for females, who accounted for 49.0% of the denominator population (eTable 2 in Supplement 1). The mean (SD) age at the time of the event was 15.4 (2.0) years across all years (Table 1). Of all events, 19.4% were direct inpatient admissions and 80.6% were ED presentations; 44.4% of ED presentations resulted in inpatient care. Suicidal ideation accounted for most cases (74.1%); the rate of suicidal ideation was even higher among those directly admitted to inpatient care (94.0%). Allowing multiple mechanisms, the most commonly used method of suicide attempt was drug poisoning (74.5%), followed by sharp objects (10.6%) and nondrug poisoning (5.6%) (eTable 3 in Supplement 1). Drug poisoning occurred less among males than females (65.9% vs 77.3%; *P* < .001) but nondrug poisoning occurred more among males than females (8.0% vs 4.9%; *P* < .001). Drug poisoning occurred less among children aged 10 to 12 years than adolescents aged 13 to 18 years (53.4% vs 75.6%; *P* < .001), while sharp objects were used more often by children aged 10 to 12 years than adolescents aged 13 to 18 years (15.1% vs 10.4; *P* < .001%).

**Table 1.** Patient Characteristics of ED Visits and Hospitalizations for Suicidal Ideation and Suicide Attempts Among Children and Adolescents, 2016-2021

Characteristic	Patients, No. (%)				*P* value^a^
	2016-2021 (N = 73 123)	2016-2019 (n = 48 425)	2020 (n = 11 301)	2021 (n = 13 397)	
Female sex	48 349 (66.1)	31 144 (64.3)	7682 (68.0)	9523 (71.1)	<.001
Male sex	24 774 (33.9)	17 281 (35.7)	3619 (32.0)	3874 (28.9)	
Age, mean (SD), y	15.4 (2.0)	15.4 (2.0)	15.3 (2.0)	15.2 (1.9)	<.001
Age, y					
10	1011 (1.4)	711 (1.5)	149 (1.3)	151 (1.1)	<.001
11	1830 (2.5)	1240 (2.6)	284 (2.5)	306 (2.3)	<.001
12	3767 (5.2)	2346 (4.8)	651 (5.8)	770 (5.7)	<.001
13	6568 (9.0)	4031 (8.3)	1095 (9.7)	1442 (10.8)	<.001
14	9513 (13.0)	6070 (12.5)	1535 (13.6)	1908 (14.2)	<.001
15	12 430 (17.0)	8069 (16.7)	1926 (17.0)	2435 (18.2)	<.001
16	13 442 (18.4)	9050 (18.7)	2004 (17.7)	2388 (17.8)	<.001
17	13 445 (18.4)	9176 (18.9)	1986 (17.6)	2283 (17.0)	<.001
18	11 117 (15.2)	7732 (16.0)	1671 (14.8)	1714 (12.8)	<.001
Region					
Northeast	5584 (7.6)	3627 (7.5)	835 (7.4)	1122 (8.4)	<.001
Midwest	24 429 (33.4)	16 049 (33.1)	3831 (33.9)	4549 (34.0)	
South	26 834 (36.7)	17 803 (36.8)	4142 (36.7)	4889 (36.5)	
West	16 276 (22.3)	10 946 (22.6)	2493 (22.1)	2837 (21.2)	
Type of episodes					
Suicidal ideation	54 188 (74.1)	35 847 (74.0)	8393 (74.3)	9948 (74.3)	.79
Suicide attempts	18 935 (25.9)	12 578 (26.0)	2908 (25.7)	3449 (25.7)	
ED or inpatient					
ED	58 960 (80.6)	39 119 (80.8)	9019 (79.8)	10 822 (80.8)	.055
ED to inpatient	26 196 (44.4)	17 333 (44.3)	4379 (48.6)	4484 (41.4)	<.001
Direct inpatient	14 163 (19.4)	9306 (19.2)	2282 (20.2)	2575 (19.2)	.06
Type of episode by ED or inpatient status					
ED					
Suicidal ideation	40 874 (69.3)	27 066 (69.2)	6254 (69.3)	7554 (69.8)	.47
Suicide attempts	18 086 (30.7)	12 053 (30.8)	2765 (30.7)	3268 (30.2)	
ED to inpatient					
Suicidal ideation	17 155 (65.5)	11 342 (65.4)	2896 (66.1)	2917 (65.1)	.55
Suicide attempts	9041 (34.5)	5991 (34.6)	1483 (33.9)	1567 (34.9)	
Direct inpatient					
Suicidal ideation	13 314 (94.0)	8781 (94.4)	2139 (93.7)	2394 (93.0)	.03
Suicide attempts	849 (6.0)	525 (5.6)	143 (6.3)	181 (7.0)	

Abbreviation: ED, emergency department.

^a^ Reported for the comparison of 3 time periods: 2016 to 2019, 2020, and 2021.

### Rates of ED Visits and Hospitalizations for Suicidality

**Figure 1.**
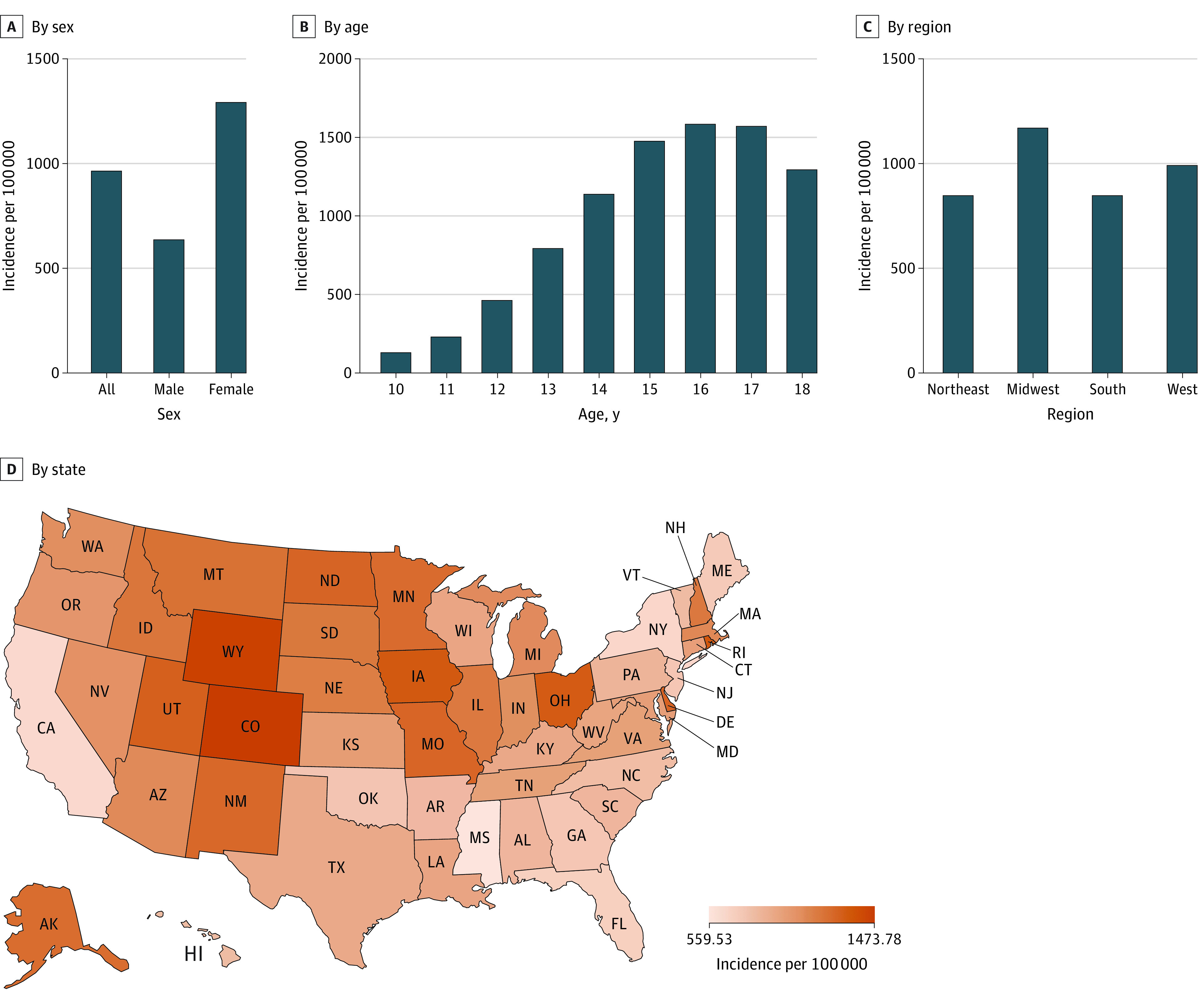
Mean Annual Rates of Emergency Department Visits and Hospitalizations for Suicidal Ideation and Suicide Attempts Among Children and Adolescents Aged 10 to 18 Years, 2016-2021 Rates were estimated using Poisson regression including age, sex, yearly trends, and geographic areas.

From 2016 to 2021, the mean annual incidence of ED visits and hospitalizations for suicidality in the US was 964 per 100 000 children and adolescents (95% CI, 956-972 per 100 000): 724 per 100 000 (95% CI, 717-731 per 100 000) for suicidal ideation and 240 per 100 000 (95% CI, 236-243 per 100 000) for suicide attempts. Rates of suicidality differed by sex, age, and geographic areas (Figure 1). Rates were found to be twice as high among females compared with males (IRR, 2.03 [95% CI, 2.00-2.06]) and exhibited an increase as ages increased until 16 to 17 years, followed by a decrease at age 18 years. When rates by region were examined, the Midwest had the highest rate at 1169 per 100 000 (95% CI, 1155-1184 per 100 000), followed by the West (991 per 100 000 [95% CI, 976-1007 per 100 000]), South (848 per 100 000 [95% CI, 838-858 per 100 000]), and Northeast (848 per 100 000 [95% CI, 825-870 per 100 000]). There were significant variations across states, ranging from 560 per 100 000 (95% CI, 495-624 per 100 000) to 1474 per 100 000 (95% CI, 1428-1520 per 100 000) (Figure 1). California, with a rate of 635 per 100 000 (95% CI, 616-655 per 100 000), had one of the lowest rates, unlike other states in the West region. Mountain states, such as Colorado and Wyoming, showed the highest rates at 1474 per 100 000 (95% CI, 1428-1520 per 100 000) and 1445 per 100 000 (95% CI, 1214-1676 per 100 000), respectively.^50,51^

### Temporal Trends of ED Visits and Hospitalizations for Suicidality

**Figure 2.**
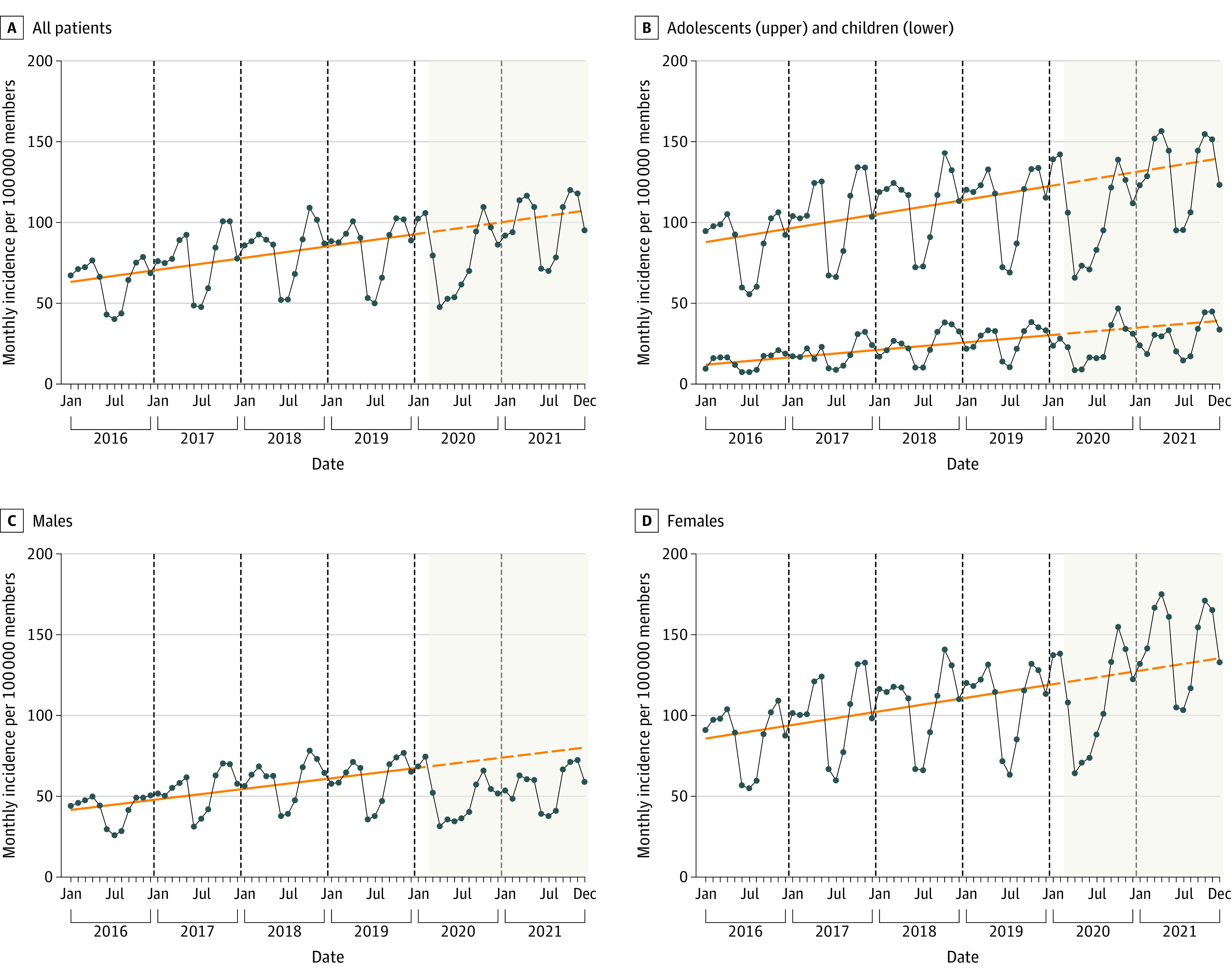
Temporal Trends of Emergency Department Visits and Hospitalizations for Suicidal Ideation and Suicide Attempts Among Children and Adolescents, 2016-2021 The shaded background indicates the COVID-19 period from March 2020 onward. Connected dots indicate the observed monthly incidence per 100 000 members, solid orange lines indicate the deseasonalized trends, and dashed orange lines depict the projected trends according to the prepandemic trend had the pandemic not occurred.

Overall annual rates increased from 760 per 100 000 (95% CI, 745-775 per 100 000) in 2016 to 1006 per 100 000 (95% CI, 988-10 024 per 100 000) in 2019, with a mean annual increase of 9.2% (IRR, 1.09 [95% CI, 1.08-1.10]). Rates temporarily decreased to 942 per 100 000 (95% CI, 924-960 per 100 000) in 2020 (IRR, 0.94 [95% CI, 0.91-0.96] in reference to 2019) but increased to 1160 per 100 000 (95% CI, 1140-1181 per 100 000) in 2021 (IRR, 1.15 [95% CI, 1.13-1.18] in reference to 2019; IRR, 1.23 [95% CI, 1.20-1.26] in reference to 2020). These trends were similar by age group but appeared to be more distinct among females (Figure 2).

### Seasonality in Suicidality

**Figure 3.**
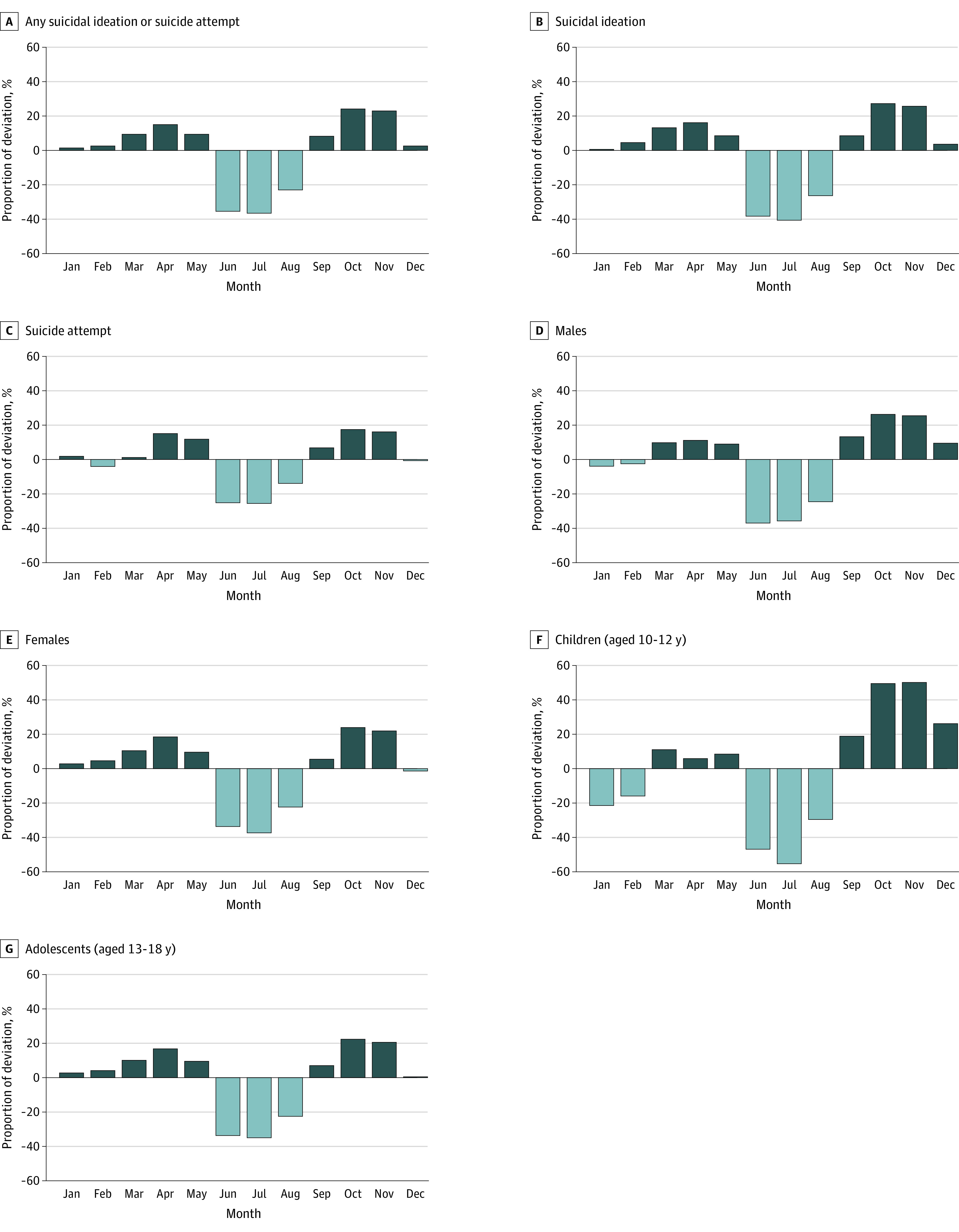
Monthly Fluctuation in Emergency Department Visits and Hospitalizations for Suicidal Ideation and Suicide Attempts Among Children and Adolescents Aged 10 to 18 Years, 2016-2019 and 2021 Fluctuations by month were expressed as percentage deviations from expected monthly incidence from Poisson regression adjusting for age, sex, census region, and annual year trends unless stratified.

The monthly fluctuations in incidence rates, expressed as a percentage deviation from the mean monthly rates, showed significant seasonal patterns (Figure 3). To quantify the seasonality in suicidality, IRRs were estimated from Poisson regressions in reference to January, which was close to the annual mean after adjusting for age, sex, region, and yearly trends (Table 2). Compared with January, the rates increased in February and reached the first peak in April, with a 15% higher rate (IRR, 1.15 [95% CI, 1.11-1.19]). Subsequently, the rates experienced a sharp decrease in June, reaching the lowest point in July, with a 37% lower rate compared with January (IRR, 0.63 [95% CI, 0.61-0.66]). The rates remained low in August and started to increase again in September, eventually reaching a second peak in October, with a 24% higher rate compared with January (IRR, 1.24 [95% CI, 1.19-1.29]). Although males showed more months of elevated suicidality during school sessions, females showed more distinct peaks in April and October (eTable 4 in Supplement 1). Age-specific seasonality increased as age increased but decreased from age 18 years, when adolescents generally completed high school (eFigure 1 in Supplement 1). From selected states, we observed consistent patterns of seasonality, with a low during summer break months and a peak in the middle of the school semester (eFigure 2 in Supplement 1).

**Table 2.** IRRs to Measure Seasonality in Suicidality

Month	Any		Suicidal ideation		Suicide attempt	
	IRR (95% CI)^a^	*P* value	IRR (95% CI)^a^	*P* value	IRR (95% CI)^a^	*P* value
January	1 [Reference]	NA	1 [Reference]	NA	1 [Reference]	NA
February	1.02 (0.98-1.06)	.39	1.04 (0.99-1.09)	.08	0.95 (0.88-1.03)	.21
March	1.09 (1.05-1.14)	<.001	1.13 (1.08-1.18)	<.001	1.00 (0.92-1.07)	.92
April	1.15 (1.11-1.19)	<.001	1.16 (1.11-1.21)	<.001	1.13 (1.05-1.22)	.001
May	1.09 (1.05-1.13)	<.001	1.08 (1.03-1.13)	.001	1.10 (1.02-1.18)	.01
June	0.65 (0.63-0.68)	<.001	0.62 (0.59-0.65)	<.001	0.74 (0.68-0.81)	<.001
July	0.63 (0.61-0.66)	<.001	0.60 (0.57-0.63)	<.001	0.74 (0.68-0.80)	<.001
August	0.77 (0.74-0.80)	<.001	0.74 (0.70-0.78)	<.001	0.85 (0.79-0.92)	<.001
September	1.07 (1.03-1.11)	<.001	1.08 (1.03-1.13)	.001	1.05 (0.98-1.13)	.19
October	1.24 (1.19-1.29)	<.001	1.27 (1.22-1.32)	<.001	1.16 (1.08-1.24)	<.001
November	1.22 (1.18-1.27)	<.001	1.25 (1.20-1.31)	<.001	1.14 (1.06-1.23)	<.001
December	1.02 (0.98-1.06)	.31	1.03 (0.99-1.08)	.16	0.99 (0.91-1.06)	.70

Abbreviations: IRR, incidence rate ratio; NA, not applicable.

^a^ The IRRs were estimated using Poisson regressions adjusting for sex, age, region, and yearly trends in reference to January 2016 to 2019 and 2021.

When we compared the monthly rates for 2019, 2020, and 2021, we noticed a disruption in the usual seasonality in 2020. We observed distinct monthly variations with clear peaks in April and October and a nadir during the summer months among children and adolescents in 2019 and 2021. However, in 2020, April and May exhibited the lowest rates, which was not observed in 2019 and 2021 (eFigure 3 in Supplement 1). As part of our sensitivity analysis, we also examined the disruptions among older adult age groups and found temporary decreases, but only in April, after which the incidence rapidly returned to normal rates (eFigure 3 in Supplement 1).

## Discussion

The present study confirms a continued upward trend in US adolescent suicidality and provides a population-level assessment using the most recent data.^5,52^ From 2016 to 2021, the mean annual incidence of ED visits and hospitalizations for suicidality was 964 per 100 000 children and adolescents (95% CI, 956-972 per 100 000) and annual incidence rates increased between 2016 and 2019, with a temporary decrease in 2020 and a return to increasing trends in 2021. The results demonstrated clear seasonal peaks in adolescent suicidality during the academic calendar. These seasonal peaks were not observed in the spring of 2020 during the COVID-19–related school closures, when event rates decreased.

Although suicidality patterns with peak rates observed in the spring are documented across the life span,^13,31,32,33,34,35,36,53^ few studies have examined the association between the risk of suicidality among children and adolescents and the academic school year. Hansen and Lang^21^ reported a marked decrease in youth suicide during months when school was not in session, ruling out other potential explanations such as economic conditions, weather, or seasonal affective disorder patterns. They suggested that youths may face increased stress and decreased mental health when school is in session. Lueck et al^19^ reported that emergency pediatric psychiatric visits for danger to self or others corresponded to times of school attendance, and Plemmons et al^5^ reported marked seasonal variation in emergency and inpatient encounters for suicidality among children and adolescents. Carbone et al^43^ showed that a seasonal trend for child and adolescent suicidality was associated with the school year, which was not present for adults. The in-school experience brings social, academic, and extracurricular stressors as well as poorer sleep habits, each of which may have detrimental outcomes for child and adolescent mental health and well-being.^29,30^ In our study, the observation of comparatively decreased suicidality during the spring 2020 COVID-19–related lockdown and subsequent return to higher rates when school resumed supports hypotheses about in-school stressors. Such rare opportunities occasioned by nationally disruptive events are not without interpretive limitations in observational studies but can help to strengthen the evidence base of plausibility even when causal inference is restricted.^37^ We cautiously interpreted the unexpected decrease in suicidality rates during the school closures in spring 2020 as further support for the association between the school calendar and suicidality among children and adolescents. In April 2020, all 3 age groups in our sensitivity analysis experienced decreases in suicidality rates, indicating the possible association of other measures, such as social distancing and the fear of contracting COVID-19, which might have deterred individuals from seeking help. However, while the older age groups returned almost to their normal ranges in May, the suicidality rates among children and adolescents remained low. This finding suggests that in addition to the broader association of various measures with suicidality rates among all age groups, the closure of schools may have been associated with a greater disruption specifically among children and adolescents.

The present results support previous work suggesting that during adolescence, females may be at greater risk for suicidality than males,^5,15,17,54,55,56^ while males are more likely to die by suicide. Our results indicated an approximately 2 times higher rate in suicidality among females compared with males and a resurgence in suicidality risk above the estimated pre–COVID-19 trend line for females once schools were reopened in late 2020.^57^ We also confirmed higher rates of suicidality in Mountain states. Prior studies found a strong positive correlation between the mean altitude of the county and the suicide rate.^50,51^ Possible reasons speculated were hypoxia affecting brain activity, greater access to firearms, and increased isolation and poverty rates. The most common method of self-harm or suicide attempt was drug poisoning among adolescents, which is consistent with increased trends in adolescent drug overdose.^58^

### Limitations

This study had several limitations. First, the present data set contained only privately insured members and may not be generalizable to all US children and adolescents. Second, the data set contained only suicidal ideation and suicide attempt and did not include data on completed suicides unless death occurred while under treatment, as data were obtained from insurance claims data from acute medical settings. In addition, the data relied on treated events as documented by claims data, leaving untreated events invisible to the analysis. Third, *ICD-10-CM* codes ranging from X71 to X83 for intentional self-inflicted injury are codes for external causes of morbidity and tend to be underreported.^59,60^ Unless a clinician is subject to a state-specific, external cause code reporting mandate or is required by a particular payer, reporting is voluntary.^61^ However, more than 90% of injury hospitalizations and ED visits are reported with an external cause code^62^; thus, the magnitude of underestimation is unknown but likely modest. Fourth, while we observed a temporary disruption in suicidality during the COVID-19 lockdowns, we cannot definitively conclude that this disruption was associated solely with school closures. There may have been other concurrent factors, such as changes in social interactions, increased stress levels, or other pandemic-related circumstances, that were associated with the observed changes in suicidality.

## Conclusions

The presence of seasonal patterns and the observed unexpected decrease in suicidality among children and adolescents after the spring 2020 COVID-19–related school closures highlight the potential association between suicidality and the school calendar. Prevention efforts can benefit by targeting periods of heightened risk.

## Notes

Supplement 1. **eTable 1.** *ICD-10-CM* Codes to Identify Suicidality **eTable 2.** Denominator Population by Age, Sex, and Region During 2016-2021 **eTable 3.** Methods of Suicide Attempt by Sex and Age Group **eTable 4.** Incidence Rate Ratios to Measure Seasonality in Suicidality by Sex and Age Group **eFigure 1.** Age-Specific Seasonality of ED Visits and Hospitalizations for Suicidal Ideation and Suicide Attempts Among Children and Adolescents During 2016-2019 and 2021 **eFigure 2.** State-Specific Seasonality in ED Visits and Hospitalizations for Suicide Ideation and Suicide Attempts Among Children and Adolescents, 10-18 Years, 2016-2019, and 2021 **eFigure 3.** Seasonality in ED Visits and Hospitalizations for Suicidal Ideation and Suicide Attempts Among Three Age Groups by Year in 2019, 2020, and 2021

Supplement 2.
Data Sharing Statement
