# Supplementary material for: Trends and Seasonality of Emergency Department Visits and Hospitalizations for Suicidality Among Children and Adolescents in the US from 2016 to 2021
Source: JAMA Netw Open. 2023 Jul 19;6(7):e2324183. doi: 10.1001/jamanetworkopen.2023.24183 (PMC10357341; doi:10.1001/jamanetworkopen.2023.24183)
Supplement: Supplement 1. — eTable 1. ICD-10-CM Codes to Identify Suicidality eTable 2. Denominator Population by Age, Sex, and Region During 2016-2021 eTable 3. Methods of Suicide Attempt by Sex and Age Group eTable 4. Incidence Rate Ratios to Measure Seasonality in Suicidality by Sex and Age Group eFigure 1. Age-Specific Seasonality of ED Visits and Hospitalizations for Suicidal Ideation and Suicide Attempts Among Children and Adolescents During 2016-2019 and 2021 eFigure 2. State-Specific Seasonality in ED Visits and Hospitalizations for Suicide Ideation and Suicide Attempts Among Children and Adolescents, 10-18 Years, 2016-2019, and 2021 eFigure 3. Seasonality in ED Visits and Hospitalizations for Suicidal Ideation and Suicide Attempts Among Three Age Groups by Year in 2019, 2020, and 2021 [file jamanetwopen-e2324183-s001.pdf]

Supplementary Online Content

Kim Y, Krause TM, Lane SD. Trends and seasonality of emergency department visits and hospitalizations for suicidality among children and adolescents in the US from 2016 to 2021. *JAMA Netw Open.* 2023;6(7):e2324183.
doi:10.1001/jamanetworkopen.2023.24183

eTable 1. *ICD-10-CM* Codes to Identify Suicidality

eTable 2. Denominator Population by Age, Sex, and Region During 2016-2021

eTable 3. Methods of Suicide Attempt by Sex and Age Group

eTable 4. Incidence Rate Ratios to Measure Seasonality in Suicidality by Sex and Age Group

eFigure 1. Age-Specific Seasonality of ED Visits and Hospitalizations for Suicidal Ideation and Suicide Attempts Among Children and Adolescents During 2016-2019 and 2021

eFigure 2. State-Specific Seasonality in ED Visits and Hospitalizations for Suicide Ideation and Suicide Attempts Among Children and Adolescents, 10-18 Years, 2016-2019, and 2021

eFigure 3. Seasonality in ED Visits and Hospitalizations for Suicidal Ideation and Suicide Attempts Among Three Age Groups by Year in 2019, 2020, and 2021

This supplementary material has been provided by the authors to give readers additional information about their work.

© 2023 Kim Y et al. *JAMA Network Open.*

eTable 1. *ICD-10-CM* Codes to Identify Suicidality

Category	***ICD-10-CM* Codes**
Suicidal ideation	R45.851
Self-inflicted harm/Suicide attempt (“Suicide attempt”)	X71–X83, intentional self-harm due to drowning and submersion, firearms, explosive or thermal material, sharp or blunt objects, jumping from a high place, jumping or lying in front of a moving object, crashing of motor vehicle, and other specified means
	T36–T50 with the 6th character of 2 (except for T36.9, T37.9, T39.9, T41.4, T42.7, T43.9, T45.9, T47.9, and T49.9, which are included if the 5th character is 2), intentional self-harm due to drug poisoning (overdose)
	T51–T65 with the 6th character of 2 (except for T51.9, T52.9, T53.9, T54.9, T56.9, T57.9, T58.0, T58.1, T58.9, T59.9, T60.9, T61.0, T61.1, T61.9, T62.9, T63.9, T64.0, T64.8, and T65.9, which are included if the 5th character is 2), intentional self-harm due to toxic effects of nonmedicinal substances
	T71 with the 6th character of 2, intentional self-harm due to asphyxiation, suffocation, strangulation
	T14.91, Suicide attempt, unknown mechanism

© 2023 Kim Y et al. *JAMA Network Open.*

eTable 2. Denominator Population by Age, Sex, and Region During 2016-2021

Year	2016	2017	2018	2019	2020	2021
**Total, n (%)**	1,297,443	1,340,791	1,315,857	1,261,271	1,178,261	1,125,679
Male	661,721 (51.0)	683,128 (50.9)	670,338 (50.9)	642,832 (51.0)	600,894 (51.0)	574,785 (51.1)
Female	635,722 (49.0)	657,663 (49.1)	645,519 (49.1)	618,439 (49.0)	577,367 (49.0)	550,894 (48.9)
**Age, n (%)**						
10	139,061 (10.7)	144,694 (10.8)	139,626 (10.6)	132,011 (10.5)	121,360 (10.3)	115,901 (10.3)
11	139,587 (10.8)	144,961 (10.8)	143,685 (10.9)	136,056 (10.8)	124,772 (10.6)	117,526 (10.4)
12	141,633 (10.9)	145,015 (10.8)	144,370 (11.0)	139,545 (11.1)	129,300 (11.0)	121,123 (10.8)
13	144,483 (11.1)	147,679 (11.0)	143,979 (10.9)	140,492 (11.1)	131,566 (11.2)	125,487 (11.1)
14	143,538 (11.1)	150,181 (11.2)	146,375 (11.1)	139,799 (11.1)	133,018 (11.3)	127,236 (11.3)
15	144,735 (11.2)	149,619 (11.2)	149,041 (11.3)	141,756 (11.2)	132,665 (11.3)	129,129 (11.5)
16	148,904 (11.5)	150,277 (11.2)	147,792 (11.2)	144,587 (11.5)	134,362 (11.4)	128,369 (11.4)
17	147,750 (11.4)	154,978 (11.6)	148,509 (11.3)	143,041 (11.3)	136,700 (11.6)	129,535 (11.5)
18	147,752 (11.4)	153,387 (11.4)	152,480 (11.6)	143,984 (11.4)	134,518 (11.4)	131,373 (11.7)
**Male, Age, n (%)**						
10	70,943 (10.7)	73,755 (10.8)	71,365 (10.6)	67,474 (10.5)	62,095 (10.3)	59,570 (10.4)
11	71,258 (10.8)	73,949 (10.8)	73,214 (10.9)	69,329 (10.8)	63,683 (10.6)	60,271 (10.5)
12	72,454 (10.9)	73,768 (10.8)	73,677 (11.0)	71,111 (11.1)	66,094 (11.0)	61,828 (10.8)
13	73,871 (11.2)	75,330 (11.0)	73,106 (10.9)	71,617 (11.1)	66,934 (11.1)	64,172 (11.2)
14	72,952 (11.0)	76,664 (11.2)	74,632 (11.1)	71,123 (11.1)	67,763 (11.3)	64,731 (11.3)
15	73,867 (11.2)	76,276 (11.2)	76,045 (11.3)	72,422 (11.3)	67,402 (11.2)	65,854 (11.5)
16	75,597 (11.4)	76,547 (11.2)	75,100 (11.2)	73,735 (11.5)	68,751 (11.4)	65,287 (11.4)
17	75,237 (11.4)	78,546 (11.5)	75,773 (11.3)	72,649 (11.3)	69,803 (11.6)	66,140 (11.5)
18	75,542 (11.4)	78,293 (11.5)	77,426 (11.6)	73,372 (11.4)	68,369 (11.4)	66,932 (11.6)
**Female, Age, n (%)**						
10	68,118 (10.7)	70,939 (10.8)	68,261 (10.6)	64,537 (10.4)	59,265 (10.3)	56,331 (10.2)
11	68,329 (10.7)	71,012 (10.8)	70,471 (10.9)	66,727 (10.8)	61,089 (10.6)	57,255 (10.4)
12	69,179 (10.9)	71,247 (10.8)	70,693 (11.0)	68,434 (11.1)	63,206 (10.9)	59,295 (10.8)
13	70,612 (11.1)	72,349 (11.0)	70,873 (11.0)	68,875 (11.1)	64,632 (11.2)	61,315 (11.1)
14	70,586 (11.1)	73,517 (11.2)	71,743 (11.1)	68,676 (11.1)	65,255 (11.3)	62,505 (11.3)
15	70,868 (11.1)	73,343 (11.2)	72,996 (11.3)	69,334 (11.2)	65,263 (11.3)	63,275 (11.5)
16	73,307 (11.5)	73,730 (11.2)	72,692 (11.3)	70,852 (11.5)	65,611 (11.4)	63,082 (11.5)
17	72,513 (11.4)	76,432 (11.6)	72,736 (11.3)	70,392 (11.4)	66,897 (11.6)	63,395 (11.5)
18	72,210 (11.4)	75,094 (11.4)	75,054 (11.6)	70,612 (11.4)	66,149 (11.5)	64,441 (11.7)
**Region, n (%)**						
Northeast	113,966 (8.8)	112,816 (8.4)	114,123 (8.7)	111,052 (8.8)	105,665 (9.0)	100,619 (8.9)
Midwest	352,723 (27.2)	354,620 (26.4)	354,787 (27.0)	341,225 (27.1)	337,254 (28.6)	324,944 (28.9)
South	543,443 (41.9)	578,970 (43.2)	548,256 (41.7)	531,511 (42.1)	480,246 (40.8)	456,371 (40.5)
West	287,311 (22.1)	294,385 (22.0)	298,691 (22.7)	277,483 (22.0)	255,096 (21.7)	243,745 (21.7)

© 2023 Kim Y et al. *JAMA Network Open.*

eTable 3. Methods of Suicide Attempt by Sex and Age Group

Methods	Total, n (%) (N= 18,935)	Male vs Female			Children vs Adolescents		
		Male (n= 4,669)	Female (n= 14,266)	p-value	Children (n= 890)	Adolescents (n= 18,045)	p-value
Poisoning, drug	14,109 (74.5)	3,075 (65.9)	11,034 (77.3)	<0.001	475 (53.4)	13,634 (75.6)	<0.001
Sharp objects	2,013 (10.6)	451 (9.7)	1,562 (10.9)	0.013	134 (15.1)	1,879 (10.4)	<0.001
Poisoning, non-drug	1,068 (5.6)	375 (8.0)	693 (4.9)	<0.001	76 (8.5)	992 (5.5)	<0.001
Other mechanisms	2,106 (11.1)	783 (16.8)	1,323 (9.3)	<0.001	187 (21.0)	1,919 (10.6)	<0.001

*Percentages may not total 100 because more than one method was allowed

© 2023 Kim Y et al. *JAMA Network Open.*

eTable 4. Incidence Rate Ratios to Measure Seasonality in Suicidality by Sex and Age Group

	Male		Female		Children		Adolescents	
Month	IRR* (95% CI)	p-value	IRR* (95% CI)	p-value	IRR* (95% CI)	p-value	IRR* (95% CI)	p-value
Jan	1.00 (reference)		1.00 (reference)		1.00 (reference)		1.00 (reference)	
Feb	1.01 (0.95-1.09)	0.67	1.02 (0.97-1.07)	0.45	1.07 (0.93-1.24)	0.35	1.01 (0.97-1.05)	0.52
Mar	1.13 (1.06-1.21)	<0.001	1.07 (1.03-1.12)	0.003	1.40 (1.23-1.60)	<0.001	1.07 (1.03-1.11)	<0.001
Apr	1.15 (1.08-1.22)	<0.001	1.15 (1.10-1.21)	<0.001	1.34 (1.17-1.53)	<0.001	1.14 (1.09-1.18)	<0.001
May	1.13 (1.05-1.20)	<0.001	1.07 (1.02-1.12)	0.007	1.37 (1.20-1.57)	<0.001	1.06 (1.02-1.11)	<0.001
Jun	0.66 (0.61-0.71)	<0.001	0.65 (0.62-0.69)	<0.001	0.68 (0.58-0.80)	<0.001	0.65 (0.62-0.68)	<0.001
Jul	0.67 (0.62-0.72)	<0.001	0.62 (0.58-0.65)	<0.001	0.57 (0.48-0.68)	<0.001	0.64 (0.61-0.67)	<0.001
Aug	0.78 (0.73-0.84)	<0.001	0.76 (0.72-0.80)	<0.001	0.90 (0.77-1.04)	0.16	0.76 (0.73-0.79)	<0.001
Sept	1.17 (1.10-1.25)	<0.001	1.03 (0.98-1.08)	0.28	1.50 (1.32-1.72)	<0.001	1.04 (1.00-1.08)	0.05
Oct	1.30 (1.22-1.39)	<0.001	1.21 (1.15-1.26)	<0.001	1.89 (1.66-2.15)	<0.001	1.19 (1.15-1.24)	<0.001
Nov	1.30 (1.22-1.38)	<0.001	1.19 (1.14-1.24)	<0.001	1.90 (1.67-2.16)	<0.001	1.17 (1.13-1.22)	<0.001
Dec	1.13 (1.06-1.21)	<0.001	0.97 (0.92-1.01)	0.15	1.60 (1.40-1.82)	<0.001	0.98 (0.94-1.02)	0.25

*Incidence rate ratios (IRR) were estimated using Poisson regressions adjusting for sex, age, region, and yearly trends in reference to January, 2016-2019 and 2021.

© 2023 Kim Y et al. *JAMA Network Open.*

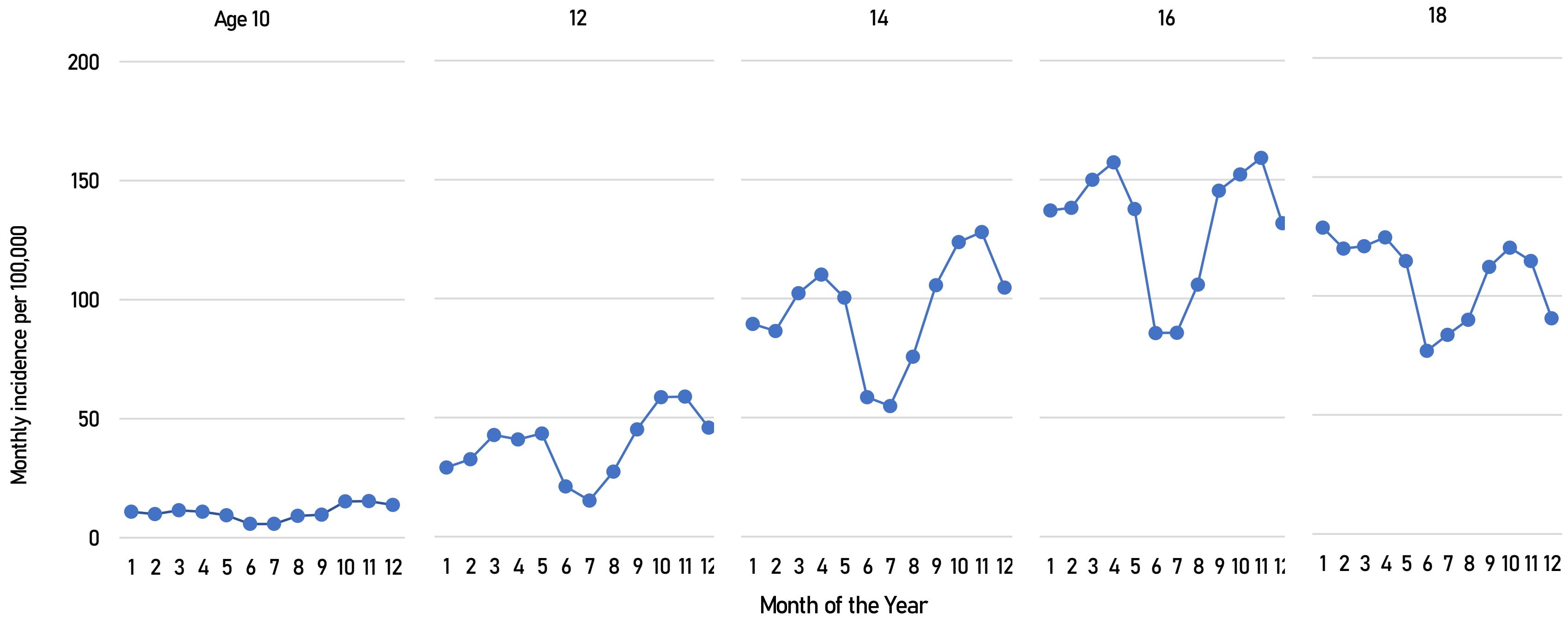

eFigure 1. Age-Specific Seasonality of ED Visits and Hospitalizations for Suicidal Ideation and Suicide Attempts Among Children and Adolescents During 2016-2019 and 2021. Monthly incidences per 100,000 members adjusted for population sex, region and year trends from Poisson regression are plotted by age. Incidences and magnitude of seasonality increase as age increases but decreases from age 18.

© 2023 Kim Y et al. *JAMA Network Open.*

Region	Division, State	Sparklines for Monthly Incidences
Northeast	New England	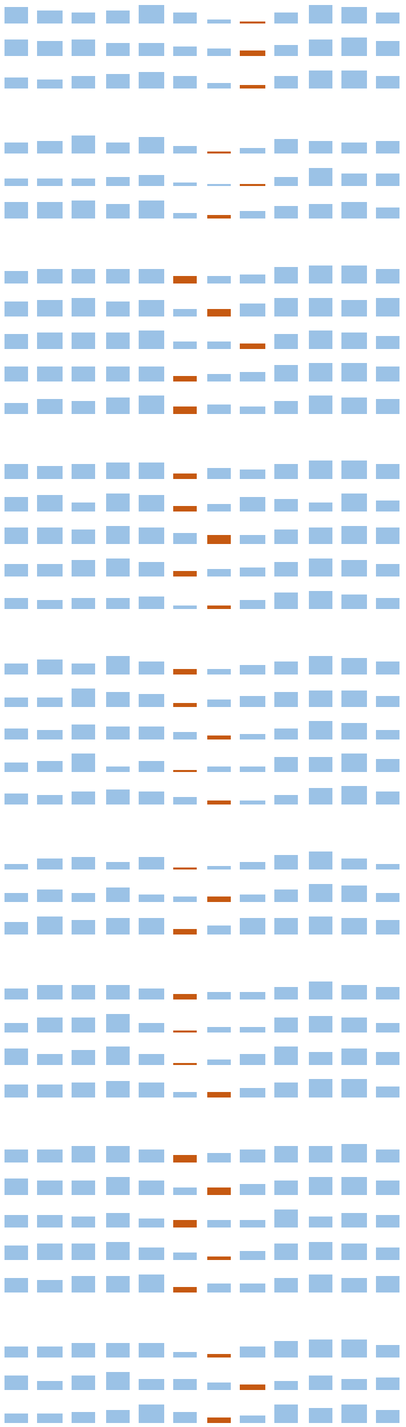
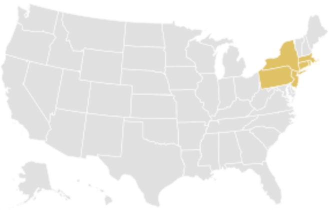	CT	
	MA	
	RI	
	Middle Atlantic	
	NJ	
	NY	
	PA	
Midwest	East North Central	
	IL	
	IN	
	MI	
	OH	
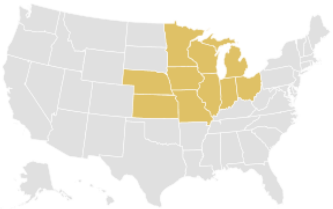	WI	
	West North Central	
	IA	
	KS	
	MN	
	MO	
	NE	
South	South Atlantic	
	FL	
	GA	
	NC	
	SC	
	VA	
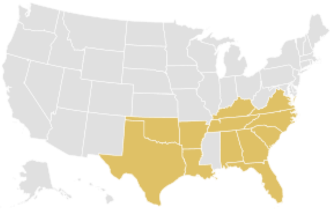	East South Central	
	AL	
	KY	
	TN	
	West South Central	
	AR	
	LA	
	OK	
	TX	
West	Mountain	
	AZ	
	CO	
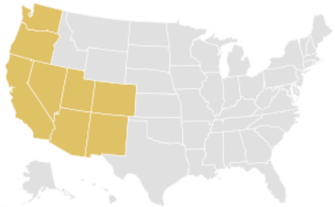	NM	
	NV	
	UT	
	Pacific	
	CA	
	OR	
	WA	

eFigure 2. State-Specific Seasonality in ED Visits and Hospitalizations for Suicide Ideation and Suicide Attempts Among Children and Adolescents, 10-18 Years, 2016-2019, and 2021. The map includes selected states in the region, excluding states with insufficient data (monthly cases 10 or less) to estimate state-specific monthly rates. Monthly incidence rates were from Poisson regressions adjusting for age, sex, and yearly trends and plotted per state using sparklines. The months of the lowest rates were colored in orange, respectively.

© 2023 Kim Y et al. *JAMA Network Open.*

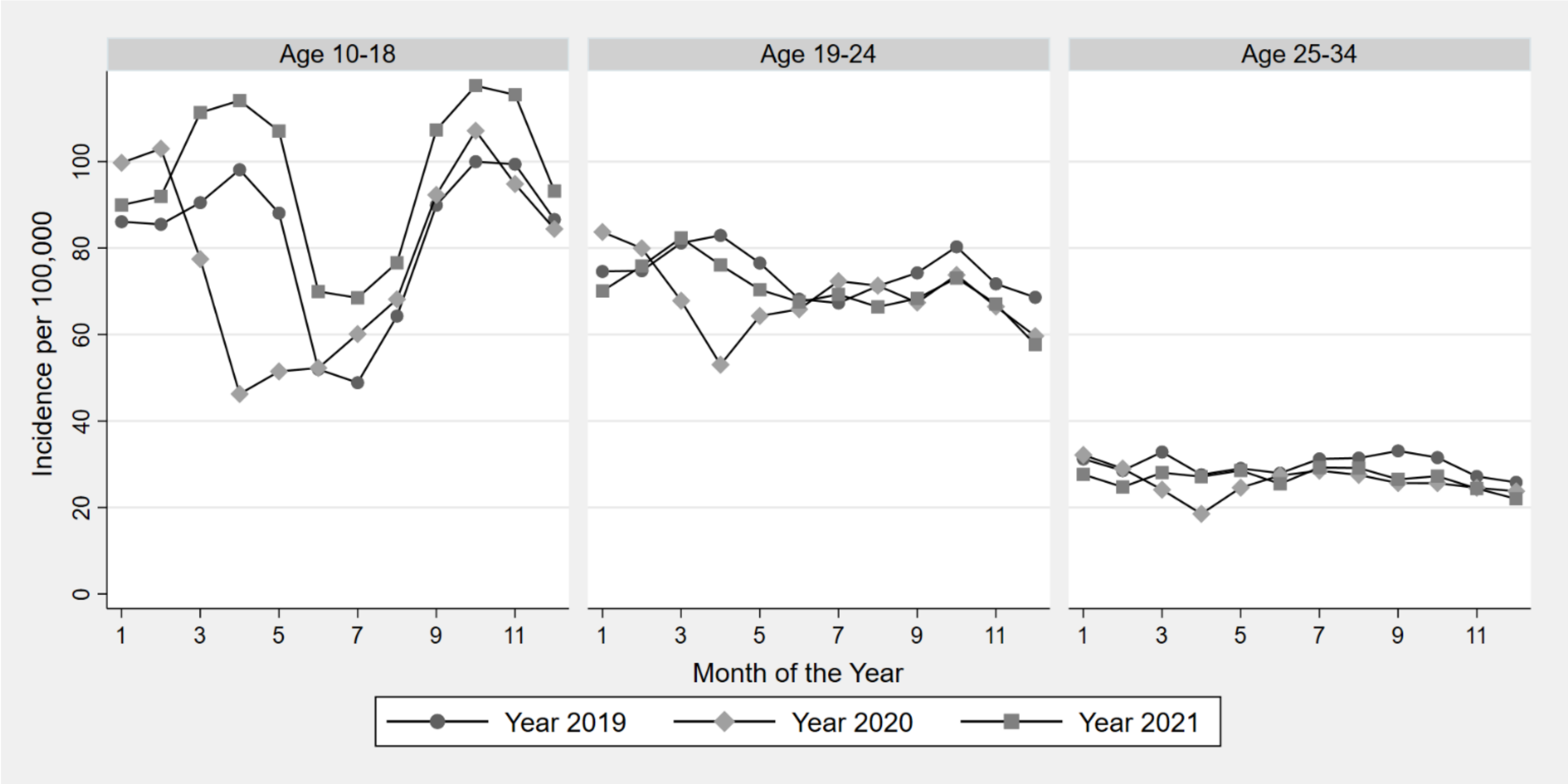

eFigure 3. Seasonality in ED Visits and Hospitalizations for Suicidal Ideation and Suicide Attempts Among Three Age Groups by Year in 2019, 2020, and 2021. Monthly incidences per 100,000 members with 95% confidence intervals adjusted for population age, sex and region from Poisson regression were plotted by year. Seasonal patterns were expected to be high in April and low in June, July, and August during summer break but in 2020 the patterns were disrupted due to school closure during the early COVID-19 pandemic.

© 2023 Kim Y et al. *JAMA Network Open.*
