# Supplementary material for: Trends and Seasonality of Emergency Department Visits and Hospitalizations for Suicidality Among Children and Adolescents in the US from 2016 to 2021
Source: JAMA Netw Open. 2023 Jul 19;6(7):e2324183. doi: 10.1001/jamanetworkopen.2023.24183 (PMC10357341; doi:10.1001/jamanetworkopen.2023.24183)
Supplement: Supplement 2. — Data Sharing Statement [file jamanetwopen-e2324183-s002.pdf]

Data Sharing Statement

Kim. Trends and Seasonality of Emergency Department Visits and Hospitalizations for Suicidality Among Children and Adolescents in the US from 2016 to 2021. *JAMA Netw Open*. Published July 19, 2023. doi:10.1001/jamanetworkopen.2023.24183

Data
Data available: No
